# Diagnostic value of ^18^F-PSMA-1007 PET/CT for predicting the pathological grade of prostate cancer

**DOI:** 10.1080/15384047.2023.2287120

**Published:** 2023-12-20

**Authors:** Xiao-Bo Niu, Yan-Peng Li, Jun Wang, Xiao-Li Mei, Xue-Yan Zhao, Ting-Ting Liu, Sha-Sha Xu, Xing-Min Han, Jing-Liang Cheng

**Affiliations:** a Department of Nuclear Medicine, The First Affiliated Hospital of Zhengzhou University, Zhengzhou, Henan, People’s Republic of China; b Department of Urinary Surgery, The First Affiliated Hospital of Zhengzhou University, Zhengzhou, Henan, People’s Republic of China; c Department of Pathology, The First Affiliated Hospital of Zhengzhou University, Zhengzhou, Henan, People’s Republic of China; d Department of Magnetic resonance, The First Affiliated Hospital of Zhengzhou University, Zhengzhou, Henan, People’s Republic of China

**Keywords:** Prostate cancer, ^18^F-PSMA-1007, PET/CT, volume-based parameters, pathological grade

## Abstract

This study was designed to evaluate the diagnostic efficacy of relevant parameters of ^18^F-prostate-specific membrane antigen (PSMA)-1007 PET/CT in predicting the pathological grade of primary prostate cancer. Briefly, a prospective analysis was performed on 53 patients diagnosed with prostate cancer by systematic puncture biopsy, followed by ^18^F-PSMA-1007 PET/CT examination prior to treatment within 10 d. The patients were grouped in accordance with the Gleason grading system revised by the International Association of Urology Pathology (ISUP). They were divided into high-grade group (ISUP 4–5 group) and low-grade group (ISUP 1–3 group). The differences in maximum standardized uptake value (SUVmax), tumor-to-background ratio (TBR), intraprostatic PSMA-derived tumor volume (iPSMA-TV), and intraprostatic total lesion PSMA (iTL-PSMA) between the high- and low-grade group were statistically significant (*p* < .001). No significant difference was found for mean standardized uptake value (SUVmean) between the high- and low-grade groups (Z =  −1.131, *p* = .258). Besides, binary multivariate logistic regression analysis showed that only iPSMA-TV and iTL-PSMA were independent predictors of the pathological grading, for which the odds ratios were 18.821 [95% confidence interval (CI): 2.040–173.614, *p* = .010] and 0.758 (95% CI: 0.613–0.938, *p* = .011), respectively. The area under the ROC of this regression model was 0.983 (95% CI: 0.958–1.00, *p* < .001). Only iTL-PSMA was a significant parameter for distinguishing ISUP-4 and ISUP-5 groups (Z =  −2.043, *p* = .041). In a nutshell, ^18^F-PSMA-1007 PET/CT has good application value in predicting the histopathological grade of primary prostate cancer. Three-dimensional volume metabolism parameters iPSMA-TV and iTL-PSMA were found to be independent predictors for pathological grade.

## Introduction

Prostate cancer, characterized with an increasing incidence year by year, is one of the most common malignant tumors in females.^1^ The biological behavior of prostate cancer varies greatly among different degrees of malignancy. High-grade prostate cancer is more prone to recurrence and distant metastasis.^2–5^ Moreover, the prognosis of advanced prostate cancer after radiotherapy is relatively poor.^6^ Nerve-sparing surgery and radiotherapy may be options in some less aggressive prostate cancers, so accurate pathological grading of prostate cancer before treatment and individualized treatment plan are of great importance to improve patient prognosis. At present, biopsy of prostate gland is the gold standard for pathologic diagnosis before treatment. However, biopsy is an invasive operation, often accompanied by hematuria, infection and other complications, causing further pain to patients.^7^ Therefore, developing an accurate noninvasive imaging method for histopathological grading is an urgent need to further obtain important information about prognosis and help design the best treatment plan for patients as early as possible.

Currently, various imaging methods have been applied in the diagnosis of prostate cancer, including pelvic magnetic resonance imaging (MRI) and transrectal ultrasound. MRI has more advantages in the application of prostate cancer diagnosis due to its better soft-tissue resolution. Although relevant sequences of MRI could be used to evaluate the pathological grade, the diagnostic criteria are still inconsistent.^8–10^ Therefore, it is of particular importance to obtain a more accurate imaging method to evaluate the histopathological grading of prostate cancer.

Prostate-specific membrane antigen (PSMA) is a type II transmembrane glycoprotein, which is lowly expressed in normal or hyperplastic prostate tissues, but highly expressed in prostate cancer.^11^ The expression of PSMA was reported to be positively correlated with the degree of malignancy, the tendency of metastasis, and the risk of early recurrence.^12,13^ In recent years, PSMA PET/CT imaging technology with PSMA as the molecular target has developed rapidly. On the one hand, it has high diagnostic efficiency for the primary and metastatic lesions of prostate cancer; on the other hand, it can detect the lesions earlier than traditional imaging examination.^14,15^ High PSMA uptake in the primary prostate cancer site ensures that its margins are easily distinguishable from normal prostate tissue with low uptake.^16,17^

There are numerous studies that the PET-CT-derived semi-quantitative parameters maximum standardized uptake value (SUVmax), PSMA-derived tumor volume (PSMA-TV), and total lesion PSMA (TL-PSMA) are valuable diagnostic indicators in the staging, evaluation of post-treatment response, and prognosis of prostate cancer.^18,19^ These diagnostic indicators were also found to be correlated with serum prostate-specific antigen (PSA) levels and Gleason scores.^20^ Considering that Gleason score is an important predictor of pathological grade, the relevant semi-quantitative parameters of PSMA PET/CT may be potentially valuable for evaluating the pathological grading of prostate cancer. Currently, SUVmax is the most commonly used parameter in PET/CT to evaluate the malignancy of prostate cancer. The three-dimensional volume parameters of ^68^Ga PSMA PET/CT, such as PSMA-TV and TL-PSMA, can more accurately reflect the tumor load of patients with prostate cancer.^19^

Nowadays, most studies focus on the accurate staging, detection of biochemical recurrence, and evaluation of efficacy or prognosis of prostate cancer.^21–23^ Reports that accurately evaluate the pathological grading of prostate cancer before treatment are rare, and the diagnostic efficacy is also not consistent. In addition, ^68^Ga-PSMA PET/CT was applied in most studies, whereas ^18^F-PSMA-1007 PET/CT was rarely used. Therefore, this research was recruited to explore the effectiveness of semi-quantitative parameters based on ^18^F-PSMA-1007 PET/CT noninvasive imaging methods in differentiating the pathological grade of prostate cancer. These semi-quantitative parameters included SUVmax, mean standardized uptake value (SUVmean), tumor-to-background ratio (TBR), intraprostatic PSMA-TV (iPSMA-TV), and intraprostatic TL-PSMA (iTL-PSMA). Also, a binary multivariate logistic regression model is also established to evaluate the diagnostic efficacy of ^18^F-PSMA-1007 PET/CT in predicting the pathological grade of prostate cancer.

## Results

### Patients’ characteristics

The mean age of the 53 patients was 66.66 ± 7.445 y, and the median serum PSA value before the PET/CT scan was 22.64 ng/ml (IQR: 17.59–37.57 ng/ml). Among the International Association of Urology Pathology (ISUP) groups, group 1 had three cases (5.7%), group 2 had 11 cases (20.8%), group 3 had 11 cases (20.8%), group 4 had 12 cases (22.6%), and group 5 had 16 cases (30.1%). The patients were divided into high-grade group (28/53, 52.8%, ISUP 4–5) and low-grade group (25/53, 47.2%, ISUP 1–3), and their mean ages were 66.43 ± 8.244 and 66.92 ± 6.595 y, respectively, with no statistical difference (*t* = −0.238, *p* = .813). Additionally, the median PSA values of the two groups were 30.18 (IQR: 18.29–53.19 ng/ml) and 18.85 ng/ml (IQR: 16.71–26.94 ng/ml), respectively, and the difference was statistically significant (z = −2.53, *p* = .011, Table 1).

**Table 1.** Comparisons of each parameter between ISUP (1–3) and ISUP (4–5).

Characteristic	Grouping		Total	Statistical Magnitude	P value
M (P25,P75)	ISUP (1–3)	ISUP (4–5)			
Age (y)	66.92 ± 6.595	66.43 ± 8.244	66.66 ± 7.445	t = −0.238	.813
PSA^a^ (ng/ml)	18.85 (IQR: 16.71–26.94)	30.18 (IQR: 18.29–53.19)	22.64 (IQR: 17.59–37.57)	Z = −2.53	.011
SUVmax^b^	9.93 (IQR: 8.79–10.67)	12.90 (IQR: 10.42–16.27)	10.65 (IQR: 9.77–13.56)	z = −3.724	<.001
SUVmean^c^	5.23 (IQR: 4.37–6.13)	6.00 (IQR: 4.35–7.13)	5.31 (IQR: 4.39–6.60)	z = −1.131	.258
TBR^d^	11.91 (IQR: 10.33–12.74)	15.27 (IQR: 12.40–19.60)	12.41 (IQR: 11.43–16.44)	Z = −3.955	<.001
iPSMA-TV^e^ (cm^−3^)	9.67 (IQR: 7.82–10.20)	14.30 (IQR: 12.27–23.06)	12.26 (IQR: 9.46–14.99)	Z = −5.060	<.001
iTL-PSMA^f^ (cm^−3^)	47.25 (IQR: 38.06–61.02)	82.43 (IQR: 58.84–143.66)	61.05 (IQR: 44.29–92.03)	Z = −4.045	<.001

^a^for prostate-specific antigen, ^b^ for maximum-standardized uptake value, ^c^ for mean-standardized uptake value, ^d^ for tumor to background ratio, ^e^ for intraprostatic PSMA-derived tumor volume and ^f^ for intraprostatic total lesion PSMA.

### Correlation and difference analysis

**Figure 1.**
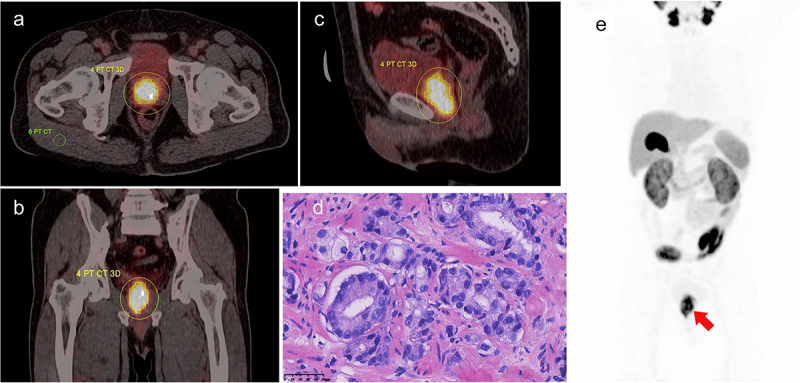
A 69-y-old male with prostate adenocarcinoma (International Society of Urological Pathology grade 5; Gleason Score: 4 + 5 = 9; PSA = 45.33 ng/ml), with ^18^F-PSMA-1007 PET/CT images depicting manually drawn VOI of major lesion in three planes and a two-dimensional ROI in the right gluteus maximus. A. Axially fused PET/CT, SUVmax: 14.47, SUVmean: 7.37, iPSMA-TV: 20.73 cm^3^, iTL-PSMA: 152.78 cm^3^, TBR: 17.43. The concentration of the imaging agent was significant at the prostate cancer site. B. Coronally fused PET/CT. C. Sagittal fused PET/CT. D. Pathological images, HE staining (×400); microscopically, small and irregular glands were observed, with local glandular fusion, co-wall, and locally scattered individual tumor cells. E. Maximum intensity projection(MIP) image.

**Figure 2.**
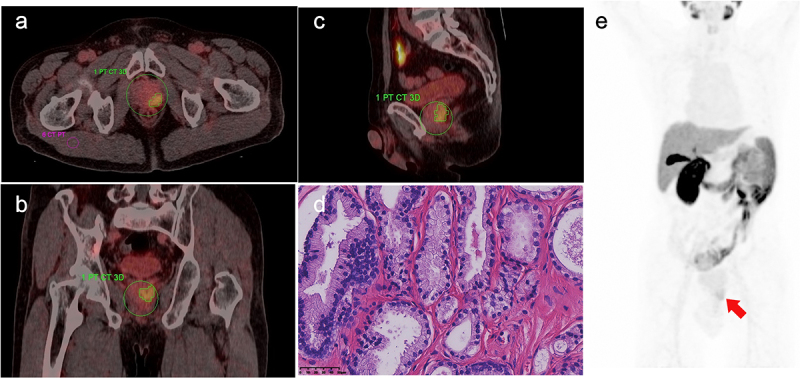
A 67-y-old male with prostate acinar adenocarcinoma (International Society of Urological Pathology grade 1; Gleason Score: 3 + 3 = 6; PSA = 8.78 ng/ml), with ^18^F-PSMA-1007 PET/CT images depicting manually drawn volume of interest (VOI) for major lesion in three planes and a two-dimensional ROI in the right gluteus maximus. A. Axially fused PET/CT, SUVmax: 5.75, SUVmean: 3.42, iPSMA-TV: 6.45 cm^3^, iTL-PSMA: 22.06 cm^3^, TBR: 6.85. There was a mild concentration of the imaging agent at the prostate cancer site. B. Sagittal fused PET/CT. C. Coronally fused PET/CT. D. Pathological images, HE staining (×400); gland presented acinous, with different sizes and shapes, and single glandular duct arrangement, and the cytoplasm was slightly dichroic. E. MIP image.

The differences in SUVmax, TBR, iPSMA-TV, and iTL-PSMA between the high- and low-grade groups were statistically significant (*p* < .001). However, no significant difference was observed for the SUVmean between the two groups (z = −1.131, *p* = .258, Table 1). The ^18^F-PSMA-1007 PET/CT images of prostate cancer in different groups and the corresponding pathological images of major lesions were shown in Figures 1 and 2. Figure 1 showed ^18^F-PSMA-1007 PET/CT image for a 69-y-old male with prostate adenocarcinoma (International Society of Urological Pathology grade 5; Gleason Score: 4 + 5 = 9; PSA = 45.33 ng/ml); and the pathological image with HE staining (×400) showed that small and irregular glands could be observed microscopically, with local glandular fusion, co-wall, and locally scattered individual tumor cells. Figure 2 displayed ^18^F-PSMA-1007 PET/CT image for a 67-y-old male with prostate acinar adenocarcinoma (International Society of Urological Pathology grade 1; Gleason Score: 3 + 3 = 6; PSA = 8.78 ng/ml); and the pathological image with HE staining (×400) exhibited acinous glands with different sizes and shapes, single glandular duct arrangement, and slightly dichroic cytoplasm, microscopically.

The correlation analysis results showed that SUVmax, SUVmean, TBR, iPSMA-TV, and iTL-PSMA were correlated with ISUP grouping (r_s_ = 0.610, 0.301, 0.635, 0.730, and 0.660, respectively; *p* < .05). Furthermore, SUVmax, SUVmean, TBR, iPSMA-TV, and iTL-PSMA were associated with serum PSA level (r_s_ = 0.519, 0.449, 0.488, 0.493, and 0.559, respectively; *p* < .01; Table 2). The binary multivariant logistic regression model analysis presented that only iPSMA-TV and iTL-PSMA were independent predictors of pathological grading, for which the odds ratios were 18.821 [95% confidence interval (CI): 2.040–173.614, *p* = .010] and 0.758 (95% CI: 0.613–0.938, *p* = .011), respectively.

**Table 2.** Correlation analysis of volumetric ^18^F-PSMA-1007 PET parameters and intensity of tracer uptake with ISUP grouping and PSA level.

Parameter	SUVmax^b^	SUVmean^c^	TBR^d^	iPSMA-TV^e^(cm^−3^)	iTL-PSMA^f^(cm^−3^)
ISUP grouping	0.610	0.301	0.635	0.730	0.660
P value	*P* < .001	*P* = .029	*P* < .001	*P* < .001	*P* < .001
PSA^a^ level	0.519	0.449	0.488	0.493	0.559
P value	*P* < .001	*P* = .001	*P* < .001	*P* < .001	*P* < .001

^a^for prostate-specific antigen, ^b^for maximum standardized uptake value, ^c^for mean standardized uptake value, ^d^for tumor to background ratio, ^e^for intraprostatic PSMA-derived tumor volume and ^f^for intraprostatic total lesion PSMA.

**Figure 3.**
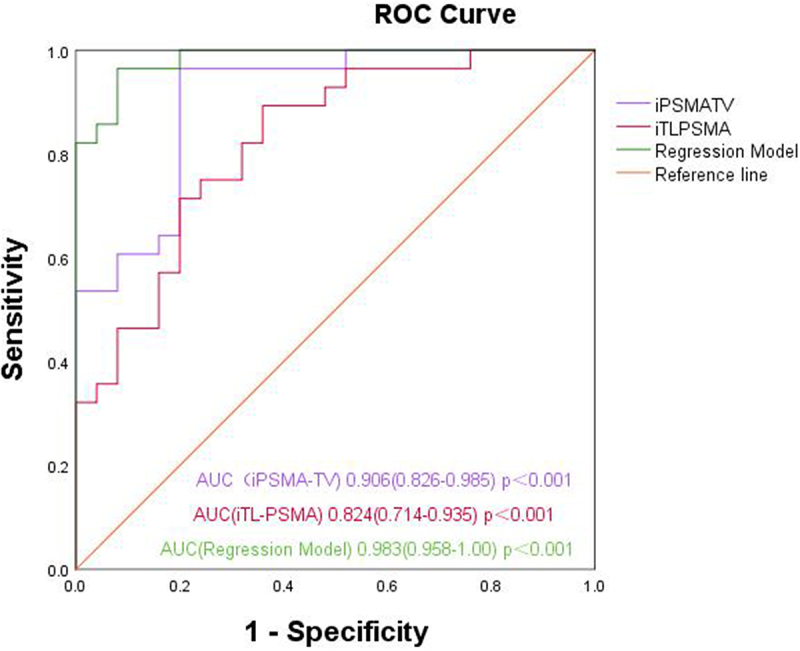
ROC curves of iPSMA-TV, iTL-PSMA and the binary multivariant logistic regression model for differentiating pathological high- and low-grade groups.

**Figure 4.**
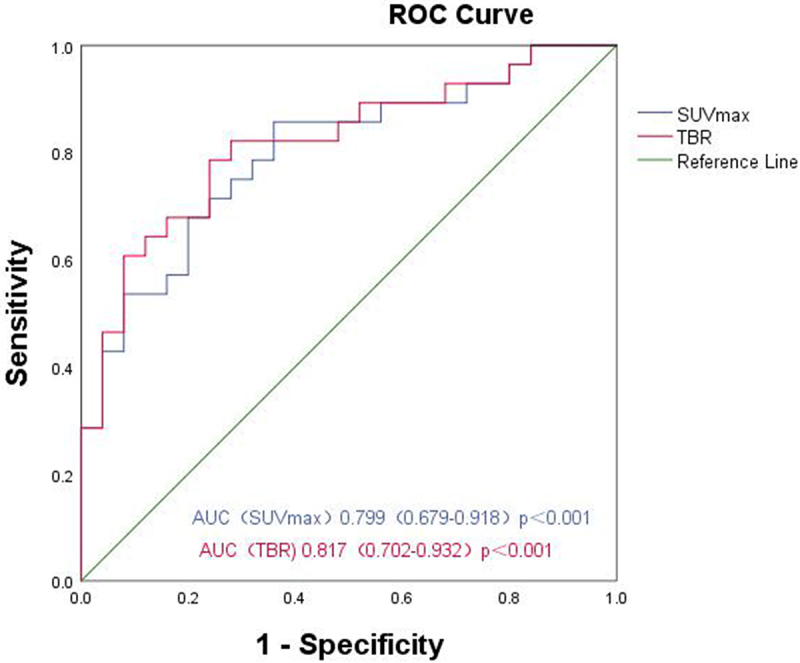
ROC curves of SUVmax and TBR for differentiating pathological high and low-grade groups.

The ROC curve was drawn in accordance with iPSMA-TV, iTL-PSMA and the prediction probability of the regression model, and the areas under the ROC curve (AUCs) were 0.906 (95% CI: 0.826–0.985, *p* < .001), 0.824 (95% CI: 0.714–0.935, *p* < .001) and 0.983 (95% CI: 0.958–1.00, *p* < .001), respectively (Figure 3). In addition, the ROC curve was used to analyze the diagnostic efficacy of TBR, and SUVmax for the pathological grading of prostate cancer; and the AUCs were 0.817 (95% CI: 0.702 − 0.932, *p* < .001), and 0.799 (95% CI: 0.679–0.918, *p* < .001), respectively (Figure 4).

Taking 10.61 cm^3^ as the cutoff value, the sensitivity and specificity of iPSMA-TV for predicting high-grade prostate cancer were determined as 96.4% and 80.0%, respectively. Using 51.42 cm^3^ as the cutoff value, the sensitivity and specificity of iTL-PSMA for predicting high-grade prostate cancer were determined to be 89.3% and 64.0%, respectively. With 12.37 as the cutoff value, the sensitivity and specificity of TBR were determined to be 78.6% and 76.0%, respectively. Taking 10.26 as the cutoff value, the sensitivity and specificity of SUVmax were 85.7% and 64.0%, respectively.

**Figure 5.**
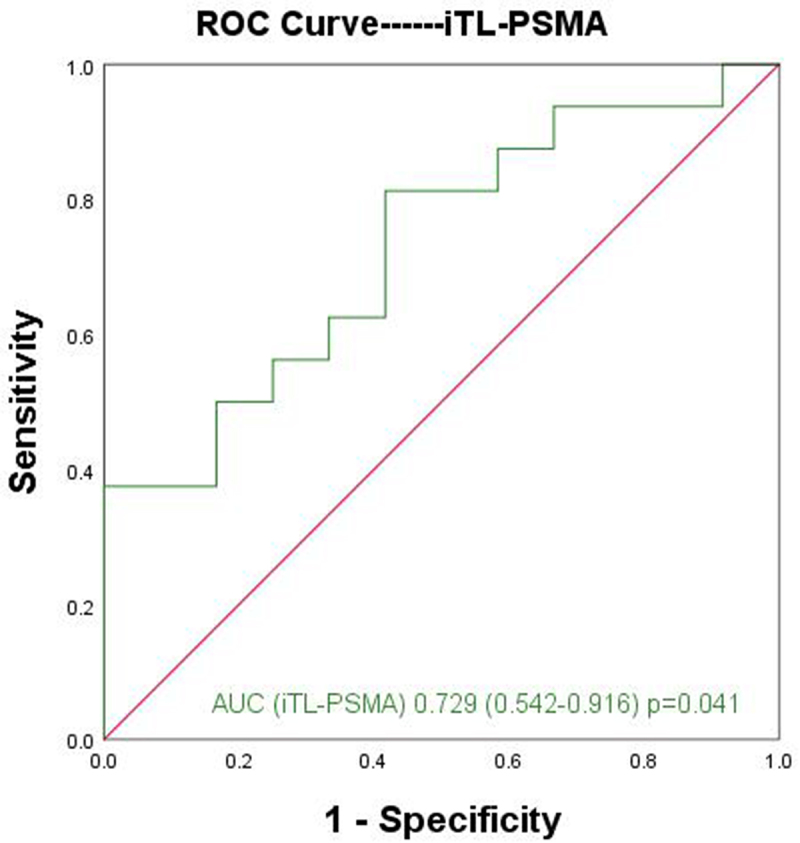
ROC curve of iTL-PSMA for differentiating ISUP-4 and ISUP-5 groups.

Furthermore, the difference of parameters between the ISUP-4 and ISUP-5 groups was analyzed. The analysis results showed that only iTL-PSMA was a significant parameter for distinguishing the two group (Z = −2.043, *p* = .041). In addition, the ROC curve was used to analyze the diagnostic efficacy of iTL-PSMA for distinguishing the ISUP-4 and ISUP-5 groups, with AUC of 0.729 (95% CI: 0.542–0.916) (Figure 5). With 71.63 cm^3^ as the cutoff value, the sensitivity and specificity of iTL-PSMA for predicting ISUP-5 group were found to be 81.3% and 58.3%, respectively. Besides, no significant difference was observed for SUV max, SUVmean, TBR, and iPSMA-TV between ISUP-4 and ISUP-5 groups (*p* > .05, Table 3).

**Table 3.** Comparisons of each parameter between ISUP-4 and ISUP-5.

Group	SUVmax	SUVmean	TBR	iPSMATV	iTLPSMA
ISUP*-5(IQR)	14.41(10.68–20.42)	6.28(4.63–9.14)	17.50(13.73–24.92)	19.61(11.82–31.10)	94.93(73.77–295.10)
ISUP*-4(IQR)	11.29(9.99–13.52)	4.84(4.19–6.61)	13.73(12.02–16.27)	13.16(12.31–15.16)	67.80(51.67–91.96)
Z	－1.718	－1.625	－1.903	－1.439	－2.043
P value	0.086	0.104	0.057	0.150	0.041

*International Society of Urological Pathology.

## Discussion

The prognosis of patients with prostate cancer is closely related to the pathological grades of prostate cancer, and ISUP grading is one of the important information of the pathologic results of prostate cancer biopsy. In this study, we investigated the diagnostic efficacy of multiple semi-quantitative parameters of ^18^F-PSMA-1007 PET/CT for the pathological grading of prostate cancer. Previous findings suggested that a high proportion of Gleason grade 4 and 5 lesions may adversely affect the prognosis of the disease.^24–26^ Therefore, in the present study, ISUP-3 group was used as the boundary, and the patients were divided into high-grade group and low-grade group. Early diagnosis and accurate pathological grading of tumors are of great importance for the formulation of treatment plans and the improvement of prognosis, such as active regular follow-up, radiotherapy, endocrine therapy, and surgical excision. There were previous studies that ^68^Ga-PSMA PET/CT could be reliably used to detect prostate cancer because it could clearly show the lesion boundaries.^27,28^ However, the relatively short half-life of ^68^Ga and the high energy of positron make ^18^F-labeled radioactive tracers more widely used. ^18^F-PSMA-1007 is a new radiotracer and a second generation of radio-fluorinated glucose-ureamide PSMA inhibitor.^29^ The concentration of the imaging agent in the bladder is relatively low because only a small amount is excreted through the urinary system, and less interference exists in the prostate area. In addition, ^18^F-PSMA-1007 has a longer half-life and higher physical spatial resolution, and thus could be well used in the diagnosis and staging of prostate cancer. Kuten et al.^30^ and Giesel et al.^31^ have shown that ^18^F-PSMA-1007 possesses a high detection rate equal to or better than ^68^Ga-PSMA-11 in staging and biochemical recurrence, even in patients with PSA levels ≤0.5 ng/ml.

At present, most studies about ^18^F-PSMA-1007 PET/CT focused on the accurate staging of prostate cancer, detection of biochemical recurrence, and evaluation of prognosis or efficacy. On the contrary, there are few reports on the evaluation of tumor load or the pathological grade of primary prostate cancer by ^18^F-PSMA-1007 PET/CT, and the diagnostic efficacy is also not uniform. Hong et al.^32^ used ^18^F-PSMA-1007 PET/CT to evaluate 101 cases of pre-treatment and non-metastatic primary prostate cancer. They found that, SUVmax, as a parameter able to distinguish medium-risk prostate cancer from high-risk prostate cancer, was significantly positively correlated with Gleason score and the serum PSA level of prostate cancer.^32^ A previous studies pointed out that the 3D volume parameters ^68^Ga-PSMA PET/CT derived primary prostate tumor PSMA-TV and TL-PSMA had high sensitivity and specificity in the diagnosis of prostate cancer metastasis.^33^ In addition, PSMA-TV and TL-PSMA were correlated with serum PSA and Gleason score.^34^ Specifically, SUVmax is a marker reflecting the uptake of the imaging agent within a single voxel, whereas PSMA-TV and TL-PSMA are markers reflecting the uptake of the imaging agent for the tumor as a whole. However, the ability of different parameters to differentiate the histopathological grade of a tumor varies. In the present study, the ^18^F-PSMA-1007 PET/CT-derived parameters SUVmax, SUVmean, TBR, iPSMA-TV, and iTL-PSMA were all correlated with the ISUP classification group. Moreover, iPSMA-TV, iTL-PSMA, and TBR had a closer correlation with the ISUP classification group than SUVmax; whereas compared with SUVmax, SUVmean exhibited a weaker correlation with the ISUP classification group. In addition, statistically significant differences were observed in the SUVmax, TBR, iTL-PSMA, and iPSMA-TV between the high-grade group and the low-grade group. Liu et al.^35^ conducted a study on a group of newly diagnosed prostate cancer, and their results also showed statistically significant differences in the SUVmax, iTL-PSMA, and iPSMA-TV between the high-risk group and the low-to-medium-risk group. This finding is similar to those of the present study.

Schmuck et al.^36^ evaluated patients after treatment with ^68^Ga-PSMA I&T PET/CT and examined the correlation of parameters SUVmax, SUVmean, PSMA-TV, and TL-PSMA with serum PSA level. They confirmed that changes in PSMA-TV and TL-PSMA were significantly correlated with changes in PSA level, whereas SUVmax and SUVmean were not significantly correlated with PSA level. Also, they suggested that PSMA-TV and TL-PSMA may be helpful in monitoring the therapeutic response. Schmidkonz et al.^34^ evaluated a group of prostate cancer patients with biochemical recurrence after postoperative treatment by ^68^Ga-PSMA PET/CT. Their findings revealed that when the number of systemic positive lesions was more than one, only SUVmax, wbPSMA-TV, and wbTL-PSMA were significantly correlated with serum PSA level. The correlation between wbPSMA-TV or wbTL-PSMA and PSA level was better than that between SUVmax and PSA level. When only one positive lesion was present, SUVmax, SUVmean, wbPSMA-TV, and wbTL-PSMA were all correlated with PSA level. Furthermore, the authors pointed out that wbPSMA-TV and wbTL-PSMA could be well used to evaluate the systemic tumor load of patients. The difference between the present study and the above studies may be related to the difference between the study objects and the imaging agents. The previous studies focused on the postoperative and post-treatment responses of patients, whereas the present study reported the condition of the primary tumor in the prostate before treatment.

Gündoğan et al.^37^ used ^68^Ga-PSMA PET/CT to study liver cancer and found that the ratio of SUVmax between cancer foci and right gluteus medius muscle was a parameter with diagnostic value. In the present study, the diagnostic value of the SUVmax ratio (TBR) between the major prostatic lesions and the right gluteus maximus muscle for predicting ISUP grade was analyzed. The analysis results showed that TBR was correlated with serum PSA level and ISUP grade, and the correlation between TBR and ISUP grade was better than that between SUVmax and SUVmean but weaker than that between iPSMA-TV and iTL-PSMA. Therefore, TBR is a parameter of high diagnostic value for evaluating the pathological grade of prostate cancer, and its diagnostic efficacy is superior to that of SUVmax.

Liu proposed that iTL-PSMA was the most effective parameter in the diagnosis of high-risk prostate cancer; the specificity of iTL-PSMA and iPSMA-TV was higher than the results of the present study, the sensitivity of iTL-PSMA and iPSMA-TV was lower, the sensitivity of SUVmax was higher, and the specificity was lower.^35^ These differences may be related to different imaging agents and the selection of cases.

Makino et al.^38^ stated that GS9–10 (ISUP-5) prostate cancer has worse prognosis than GS8 (ISUP-4) prostate cancer. Therefore, the difference for the parameters between the ISUP-4 and ISUP-5 groups was analyzed, and the analysis results showed that only iTL-PSMA was a significant parameter for distinguishing the two groups. In addition, the ROC curve was used to analyze the diagnostic efficacy of iTL-PSMA for distinguishing the ISUP-4 and ISUP-5 groups, and the AUC was 0.729. Taking 71.63 cm^3^ as the cutoff value, the sensitivity and specificity of iTL-PSMA for predicting ISUP-5 group were 81.3% and 58.3%, respectively. On the contrary, no significant difference was observed for SUV max, SUVmean, TBR, and iPSMA-TV.

There are certain limitations in this study. Given that the sample size is small, the non-uniform diagnostic effectiveness of the parameters still needs to be confirmed by more prospective studies with a larger sample size. Also, the relationship between the quantitative parameters of ^18^F-PSMA-1007 PET/CT and pathological grade and prognosis still requires to be further verified. In addition, the ^18^F-PSMA-1007 PET/CT-derived volume-based parameters iPSMA-TV and iTL-PSMA showed better diagnostic value than SUVmax in this study. However, due to the inherent measurement errors of these parameters, point-to-point control studies between volume-based 3D PET/CT imaging and pathology studies are needed to establish accurate and stable quantitative measurements of lesions.

## Conclusion

To sum up, ^18^F-PSMA-1007 PET/CT has good application value in predicting the histopathological grade of primary prostate cancer. To be specific, three-dimensional volume metabolism parameters iPSMA-TV and iTL-PSMA were independent predictors for pathological grade; iTL-PSMA was a significant parameter for distinguishing the ISUP-4 and ISUP-5 groups. Besides, three-dimensional metabolic parameters have more advantages than conventional metabolic parameters. For one thing, the semi-quantitative parameters SUVmax, SUVmean, TBR and the volume-based parameters iPSMA-TV and iTL-PSMA were all correlated with pathological grading and pre-treatment PSA level. For another, the diagnostic efficiency of TBR was better than that of SUVmax. Finally, the binary multivariant logistic prediction model established in this study is a reliable model for the identification of ISUP high- and low-grade groups.

## Methods

### Patients

A total of 53 newly diagnosed patients with biopsy-confirmed prostate cancer were included between June 2020 and October 2022. This study was approved by the Ethics Committee of First Affiliated Hospital of Zhengzhou University (permit 2020-KY-346). The inclusion criteria were listed as follows: prostate cancer patients (1) were diagnosed by biopsy; (2) signed informed consent; and (3) were willing to accept follow-up. The exclusion criteria were shown as follows: patients who had received local or systemic treatment or patients with tumors at other sites. All 53 patients underwent ultrasound-guided targeted puncture biopsy, and histopathology corresponding to major lesions was selected as the golden standard.

The patients were grouped in accordance with the Gleason grading system revised by the ISUP.^39^ If there are two or more Gleason scores corresponding to the biopsy pathology of the main lesion area, the higher score is selected as the basis for pathological grading. Patients were divided into high-grade group (ISUP 4–5 group) and low-grade group (ISUP 1–3 group). Besides, written informed consent was obtained from all individual participants included in the study.

### Radiopharmaceutical

^18^F-PSMA-1007 precursor, cassettes, and reagents for the synthesis of ^18^F-PSMA-1007 were obtained from ABX Advanced Biochemical Compounds (Radeberg, Germany). Specifically, ^18^F-PSMA-1007 was synthesized using the chemical method of HM-20 cyclotron and CFN-100 synthesis module from Sumitomo Corporation (Japan). The radiotracer was obtained in over 98% radiochemical purification yield by radio-thin-layer chromatography and high-performance liquid chromatography analysis. All products were prepared by excellent technology under aseptic and pyrogen-free conditions.

### Imaging protocol

All patients underwent ^18^F-PSMA-1007 PET/CT examination within 10 d after diagnosis of prostate cancer, and they had not received any treatment prior to the examination. ^18^F-PSMA-1007 was intravenously injected into each patient at 3.7 MBq/kg (median activity: 261.6 MBq; range: 186.3–321.7 MBq), and PET and CT scans were performed after 60 ± 10 min. ^18^F-PSMA-1007 images were acquired from a body PET/CT scanner [Biograph TruePoint64 (52) ring, Siemens, Germany]. The CT scans were performed from head to the middle thigh, followed by the PET acquisition. The CT acquisition and reconstruction parameters were shown as follows: CT voltage of 120 keV, current of 100 mAs, pitch of 0.8 mm, tube single-turn rotation time of 0.5 s, and scanning layer thickness of 3 mm. A three-dimensional mode was used to obtain PET images with the following parameters: matrix of 168 × 168 and slice thickness and interval of 3–5 mm. A total of 8–10 beds were scanned by PET, and the emission scan time for each bed position was 1.5 min. After attenuation correction of PET images with CT data, whole-body (wb) PET and CT images were obtained by iterative reconstruction.

### Image analysis

All ^18^F-PSMA-1007 PET/CT images were analyzed using a dedicated workstation (Syngo, Siemens), which allowed the review of PET, CT, and fused imaging data in axial, coronal, and sagittal slices. Briefly, two experienced nuclear medicine practitioners were jointly responsible for interpreting all ^18^F-PSMA-1007 PET/CT scans, as well as localization and comprehensive analysis on the original and fused images. Then, the main lesions were identified. The major lesions on PET/CT were defined as those with the highest uptake within the prostate, and the location of major lesions was evaluated in accordance with the biopsy results. The threshold method was used to delineate the volume of interest (VOI) for the major lesions with local abnormal radioactive uptake concentration in the prostate. VOI was manually adjusted to match the edges of the positive lesions. The SUVmax, SUVmean, iPSMA-TV, and other parameters in VOI were calculated automatically by the software. Next, iTL-PSMA was calculated on basis of the formula of iTL-PSMA = SUVmean × iPSMA-TV. Regarding the background, a 1.5 ± 0.2 cm^2^ two-dimensional circular area of interest was sketched in the right gluteus maximus muscle, and the SUVmax of the right gluteus maximus muscle was obtained as the background. TBR was calculated using the formula of TBR = primary lesion SUVmax÷background SUVmax. Any disagreement was resolved by consensus.

### Statistical analysis

SPSS version 26.0 statistical software (IBM Corp., Armonk, NY) was employed for data analysis. Measurement data conforming to normal distribution were represented by $\bar{X}$± s. Comparison of the mean between two independent samples conforming to normal distribution was conducted using two-independent sample t test. Measurement data with non-normal distribution were represented by M (IQR). Wilcoxon Mann–Whitney U-test was used to examine the differences in semi-quantitative PET/CT parameters among subgroups. The correlation of semi-quantitative PET/CT parameters with ISUP grouping and pre-treatment PSA value was analyzed using Spearman’s rank correlation coefficient (p). The SUVmax, TBR, and volume-based parameters of PET/CT were used to construct a binary multivariant logistic regression prediction model of pathological grading in prostate cancer. This model was used to determine independent predictors of the pathological grading of prostate cancer, and its diagnostic efficiency was evaluated by ROC curve. In addition, the ROC curves of SUVmax, TBR, iPSMA-TV, and iTL-PSMA were separately drawn, and their diagnostic ability for the pathological grading of prostate cancer was analyzed using AUC. The cutoff value of each parameter was obtained from the Youden index, which was used as the standard to test the diagnostic accuracy of each parameter. For all statistical parameters, *p* < .05 were considered statistically significant.

## Research data

All data generated or analyzed during this study are included in this published article (and its supplementary information files).

## Trial registration

This study has been registered in the Chinese Clinical Trial Registry, a primary registry of International Clinical Trial Registry Platform, World Health Organization, under registration number ChiCTR2200059170.
